# Successful Pregnancy Post-Kidney Transplant: A Case Report

**DOI:** 10.7759/cureus.104884

**Published:** 2026-03-09

**Authors:** Nigar Mehtiyeva, Shalini Fernandes, Abeer Nouman, Komal Hazari, Laila Alhubaishi

**Affiliations:** 1 Obstetrics and Gynecology, Dubai Health, Dubai, ARE; 2 Obstetrics and Gynecology, Latifa Hospital, Dubai, ARE; 3 Obstetric Medicine, Dubai Health, Dubai, ARE

**Keywords:** fetotoxicity, highrisk pregnancy, immunosuppression, • kidney transplantation, multidisciplinary approach, postpartum

## Abstract

Pregnancy after renal transplantation is now more feasible, with documented rates of successful live births. However, pregnancy after renal transplantation is associated with an increased risk of maternal complications, including preeclampsia, graft dysfunction, hypertension, and diabetes, as well as adverse perinatal outcomes such as preterm birth and intrauterine growth restriction. Effective care requires a multidisciplinary approach. This report discusses a case of a primigravida patient who successfully delivered at term three years after kidney transplantation, with an emphasis on the clinical decision-making process, challenges, and strategies used to achieve the best possible outcome.

We present a 27-year-old female, gravida 1 para 0 (G1P0), with a history of end-stage renal disease secondary to glomerulonephritis, who underwent a renal transplant at age 24. She was maintained on tacrolimus, azathioprine, and prednisolone and had stable graft function throughout follow-up. She also had type 2 diabetes diagnosed after transplantation, which was managed with insulin. After spontaneous conception, she was followed in a high-risk antenatal clinic with joint nephrology and obstetrics oversight. Antenatal care included biweekly monitoring of renal function, including serum creatinine, estimated glomerular filtration rate (eGFR), and electrolytes, therapeutic drug monitoring of tacrolimus trough levels, frequent blood pressure assessments, and fetal surveillance using serial ultrasounds and non-stress testing.

Preeclampsia prophylaxis with low-dose aspirin (150 mg) was initiated at the initial antenatal visit. Blood pressure was managed with labetalol, and insulin therapy was continued with input from endocrinology. Immunosuppressive drug doses were carefully adjusted to maintain therapeutic levels. Renal function at 20 weeks was within normal range (creatinine: 0.88 mg/dL, eGFR: >80 mL/min/1.73 m²) with no evidence of graft dysfunction or proteinuria. The remainder of the pregnancy was uneventful until 36+6 weeks, when there was a mild but sustained rise in serum creatinine (1.1 mg/dL) and urea, suggesting early renal stress. After consultation with nephrology, labor was induced at 37 weeks. Stress-dose intravenous hydrocortisone was administered, and a vacuum-assisted vaginal delivery was performed. The neonate weighed 3.39 kg and did not require NICU admission. Postpartum, she experienced an atonic postpartum hemorrhage of approximately 800 mL, which was managed with uterotonic agents and close observation. Renal function stabilized within one week. Immunosuppressants were resumed at their pre-pregnancy doses, and insulin therapy was converted to metformin following endocrinology reassessment.

This report highlights the importance of individualized and multidisciplinary care in managing pregnancies among kidney transplant patients. Given the high risk of complications, including preeclampsia (up to 30%) and preterm delivery (50-60%), personalized management strategies are essential. Critical elements include maintaining stable graft function before conception, optimizing immunosuppressive therapy, effectively managing diabetes and hypertension, and detecting changes in renal function early to allow timely delivery.

## Introduction

Kidney transplantation has transformed the prognosis for individuals with end-stage renal disease, improving fertility in many women with chronic kidney disease and allowing them to conceive and carry pregnancies to term [1]. Despite these advancements, pregnancy in kidney transplant recipients remains high-risk and requires careful management and close monitoring because of potential complications such as graft dysfunction or rejection, miscarriage, fetal growth restriction, preeclampsia, preterm birth, and neonatal death [2]. Nevertheless, successful pregnancy outcomes are being reported more frequently, reflecting improvements in clinical management and patient selection [1,2].

The live birth rate for pregnancies after transplantation can be favorable; however, these pregnancies are often associated with complications such as hypertension and gestational diabetes, which occur more frequently than in the general population [2]. Therefore, comprehensive preconception counseling is essential to optimize maternal health and ensure stable graft function before attempting to conceive [3]. The need for a multidisciplinary approach involving nephrologists and obstetricians is essential to address the complex physiological changes and potential risks that may occur during pregnancy in this population [4].

This case report describes the clinical journey of a 27-year-old female who successfully conceived and delivered a healthy infant three years after a living donor kidney transplant, exceeding the commonly recommended minimum interval between transplantation and pregnancy. This report emphasizes the importance of preconception planning, multidisciplinary coordination, and individualized care with ongoing monitoring to achieve favorable maternal and neonatal outcomes. By sharing this clinical experience, we aim to contribute to the growing body of literature on post-transplant pregnancy, with particular emphasis on sustained graft stability throughout gestation and the importance of timely multidisciplinary decision-making regarding labor induction to preserve maternal and renal outcomes.

## Case presentation

The patient was a 27-year-old primigravida who presented for her booking visit at 20 weeks of gestation following spontaneous conception, with delayed attendance due to social circumstances. She had undergone a living donor renal transplant in 2020 for kidney failure of unknown cause, with stable graft function. At booking (20 weeks), serum creatinine was 0.88 mg/dL, with an estimated glomerular filtration rate (eGFR) of 92.9 mL/min/1.73 m². She was on immunosuppressive therapy: prednisolone 5 mg once daily, azathioprine 100 mg once daily, and tacrolimus 9 mg once daily. The cause of her kidney failure was unknown, and she had no family history of renal failure, with only a history of diabetes in both parents. She had type 2 diabetes, diagnosed three years before pregnancy, and managed with insulin (NovoRapid 6 units three times daily and Levemir 12 units at bedtime) for one year. Glycemic control was adequate (HbA1c 5.7%).

She had taken folic acid during the first trimester but not before conception. On presentation, her BMI was 26.95 kg/m², blood pressure (BP) was normal, hemoglobin was 8.7 g/dL, consistent with moderate iron-deficiency anemia, HbA1c was 5.7, and serology was negative. Renal function remained within the normal range (serum creatinine 0.88 mg/dL; reference 0.50-0.90, eGFR >90 mL/min/1.73 m²; reference >60), and no proteinuria was detected on 24-hour urine collection (0.15 g). As a follow-up plan, creatinine and eGFR were monitored biweekly to ensure renal function remained stable.

Other labs were as follows: platelets 212 ×10⁹/L (reference 150-400), aspartate aminotransferase (AST) 10 U/L (reference 0-32), alanine aminotransferase (ALT) 13 U/L (reference 0-31), bilirubin 1.0 mg/dL, and lactate dehydrogenase (LDH) 148 U/L (reference 105-222). She was followed up by nephrology and the Latifa Hospital antenatal clinic, and both the anomaly scan and follow-up scan were normal. She received extensive education on the signs and symptoms of preeclampsia, was advised to monitor her BP at home, and instructed to seek immediate medical attention if her BP became unstable or if she experienced any symptoms suggestive of preeclampsia.

Blood pressure remained well controlled without antihypertensives (<140/90 mmHg). Low-dose aspirin was started at booking (23 weeks’ gestation) for preeclampsia prophylaxis due to her high-risk status. Immunosuppressive therapy was maintained, with tacrolimus levels ranging from 3 to 6 ng/dL. She received intravenous (IV) ferosac for anemia, later transitioned to oral iron, and her hemoglobin subsequently improved to 9.6 g/dL before admission at 37 weeks. Monthly urine cultures were performed to monitor for infection. At 28 weeks, renal function remained within normal limits (creatinine 0.79 mg/dL, eGFR >60). In the late third trimester, serum creatinine increased to 1.53 mg/dL, with uric acid 10.4 mg/dL, along with a decline in platelet count to 123 ×10⁹/L and elevated LDH (306 U/L). At 37 weeks, she was admitted for induction due to worsening renal function and biochemical deterioration, raising concern for evolving graft stress and possible features on the preeclampsia spectrum. Nephrology recommended stress-dose steroids (hydrocortisone 100 mg IV three times daily) during labor and for 24 hours postpartum.

She underwent induction of labor at 37 weeks with a single dose of prostaglandin (Prostin). She received epidural analgesia, artificial rupture of membranes, IV fluids, sodium bicarbonate, and syntocinon at full cervical dilatation. Blood glucose levels were closely monitored, and the attending urologist remained on standby during the intrapartum period. She delivered at 37+1 weeks via vacuum-assisted vaginal delivery with episiotomy due to prolonged second stage and inadequate maternal expulsive effort, delivering a healthy infant (3.39 kg, Apgar scores 7 and 8). An atonic postpartum hemorrhage of approximately 800 mL occurred and was managed with bimanual uterine massage, bladder emptying, carboprost and misoprostol administration, and episiotomy repair. Cervical exploration revealed no tears. One unit of packed red blood cells (PRBC) was transfused in response to the hemorrhage. The infant remained well, was breastfed, and roomed in with the mother.

Postpartum, tacrolimus levels, renal function, and blood counts were closely monitored, while insulin was discontinued and replaced with metformin. Blood pressure normalized without medications. She was on low-molecular-weight heparin (LMWH), continued her immunosuppressive therapy, and followed a diabetic low-purine diet. At discharge, renal function was stable, and she was advised regarding contraception, nephrology follow-up, and routine postpartum review. Postpartum laboratory tests showed that renal function had returned to baseline, and she resumed her pre-pregnancy immunosuppressive regimen. Serial renal and biochemical parameters during pregnancy and postpartum are summarized in Table 1.

**Table 1:** Serial renal and biochemical lab results ^H^high; ^L^low; ^HH^markedly high Serial renal and biochemical parameters during pregnancy and postpartum, with corresponding reference ranges, demonstrating progressive renal impairment, thrombocytopenia, hyperuricemia, and elevated liver enzymes around delivery Ref. range: reference range; eGFR: estimated glomerular filtration rate; LDH: lactate dehydrogenase; AST: aspartate aminotransferase; ALT: alanine aminotransferase

Timeline	Date	Creatinine (mg/dL)	eGFR (mL/min/1.73m²)	Platelets (×10⁹/L)	Uric Acid (mg/dL)	LDH (U/L)	AST (U/L)	ALT (U/L)
Ref. range	–	0.50–0.90	>60	150–400	2.4–5.7	105–222	0–32	0–31
Booking (20w2d)	17/05/23	0.88	92.9	212	6.5ᴴ	–	10	13
Follow-up	15/08/23	1.08ᴴ	72.7	–	–	–	–	–
	28/08/23	–	–	157	–	–	–	–
	04/09/23	1.21ᴴ	63.4ᴸ	–	–	–	–	–
	08/09/23	1.48ᴴ	49.8ᴸ	–	10.4ᴴᴴ	–	–	–
Admission (37w)	11/09/23	1.53ᴴ	47.8ᴸ	–	11.2ᴴᴴ	306ᴴ	59ᴴ	58ᴴ
Delivery (day)	12/09/23	1.73ᴴ	41.3ᴸ	86ᴸ	10.9ᴴᴴ	–	–	75ᴴ
Post-delivery	13/09/23	1.33ᴴ	56.6ᴸ	–	–	509ᴴ	–	–
	14/09/23	1.07ᴴ	73.5	122ᴸ	10.2ᴴᴴ	–	41ᴴ	58ᴴ

## Discussion

Studies indicate that post-transplant pregnancies often result in live births, with rates ranging from 70% to 85% [1]. However, these pregnancies are associated with increased risks of complications, including preeclampsia (20-30%), preterm delivery (50-60%), and small-for-gestational-age infants (30-40%) [1]. In the United States, the live birth rate among kidney transplant recipients has been reported at 72.9%, higher than the 62% rate in the general population [1]. Maintenance of immunosuppressive therapy is critical to preventing graft rejection, but these medications carry teratogenic risks. Drugs such as mycophenolate mofetil (MMF) should be discontinued and replaced with safer alternatives, such as azathioprine, at least six weeks before conception [1]. Careful management of immunosuppressive therapy is essential, as certain drugs can adversely affect fetal development [3].

Pregnancy-related changes in renal physiology, including increased plasma volume, elevated glomerular filtration rate, and hormonal fluctuations, can place additional stress on a transplanted kidney. Changes in renal function occur in approximately 10-18% of pregnancies after kidney transplantation, with an increased risk of graft loss in poorly controlled cases [2]. Monitoring renal function is essential, as a transient rise in creatinine is common during the third trimester [4]. While most studies focus on short-term clinical outcomes, there is limited information on long-term maternal graft survival, long-term child neurodevelopment, patient-reported experiences, and guidelines tailored to diverse populations. The need for larger, global prospective registries is evident to better understand these specific outcomes [4].

Various factors can affect the outcome of pregnancy following kidney transplantation, including preconception kidney function, management of chronic hypertension and diabetes, risk of opportunistic infections, and the potential for obstetric complications. According to the American Society of Transplantation (AST), pregnancy is considered safe if there has been no rejection in the preceding 12 months, graft function is stable (serum creatinine <1.5 mg/dL and proteinuria <500 mg/24 hours), there are no acute infections, and immunosuppressive medication dosages are stable [3]. Preconception counseling is essential to create the most favorable conditions for pregnancy, such as maintaining stable blood pressure and kidney function [3].

Three key factors can influence pregnancy outcomes in women with transplants: preconception counseling and planning, maternal health management, and appropriate use of medications to avoid fetal toxicity. These principles were reflected in our case, where stable graft function, controlled blood pressure, and proper immunosuppression were maintained throughout pregnancy [3]. Preconception counseling is essential to reduce the risks associated with an unexpected or poorly timed pregnancy, with pregnancy recommended only at least two years post-transplantation [3]. Candidates for pregnancy should have normal blood pressure, stable serum creatinine below 1.5 mg/dL, and proteinuria under 500 mg/24 hours.

Medical management in mothers is essential for the early detection and treatment of complications such as hypertension, preeclampsia, thrombotic microangiopathy, graft dysfunction, gestational diabetes, and infection [5], as the severity of these adverse outcomes is related to the degree of kidney dysfunction. A major concern is the potential fetotoxicity of medications. Therapeutic doses of glucocorticoids and azathioprine are generally considered compatible with pregnancy, particularly after the first trimester, while calcineurin inhibitors (CNIs) are not associated with major congenital malformations but may increase the risk of prematurity and low birth weight [3]. Agents such as rituximab and eculizumab should be reserved for situations in which the potential fetal benefit outweighs the risks. In contrast, renin-angiotensin system inhibitors, mycophenolate, bortezomib, and cyclophosphamide are known to be fetotoxic and should never be prescribed during pregnancy [6].

Increased maternal age is also an important determinant of pregnancy outcomes, as older mothers face a higher risk of complications [7]. Management of gestational diabetes must also be considered, as it affects approximately 16% of pregnancies in this population [3]. Finally, the importance of a multidisciplinary approach is emphasized, with nephrologists, obstetricians, and other specialists collaborating to ensure the well-being of both mother and fetus. Such coordinated care can result in better outcomes and improved management throughout pregnancy [8].

## Conclusions

This report highlights that successful pregnancy after kidney transplantation is achievable with individualized care, stable graft function, a multidisciplinary management approach, and vigilant monitoring to ensure favorable maternal and neonatal outcomes. Key considerations include balancing immunosuppression to prevent rejection while minimizing teratogenic risks and addressing maternal complications such as hypertension, preeclampsia, and graft dysfunction. The timing of delivery in transplant recipients must be carefully planned, requiring multidisciplinary assessment, appropriate management of comorbidities, and adjustment of immunosuppressive medications throughout pregnancy. Good renal function is a critical factor for achieving positive outcomes, and regular monitoring of kidney function is essential during this period. Following pregnancy, renal function often returns to baseline in transplant recipients; however, close postpartum monitoring and adjustment of immunosuppressive therapy remain crucial.
